# Development of PAL MIND: a palliative care clinical tool to inform a pilot study

**DOI:** 10.1093/geroni/igaf122.2609

**Published:** 2025-12-31

**Authors:** Danielle Llaneza, Kristopher Kern

**Affiliations:** Rutgers Robert Wood Johnson Medical School, New Brunswick, New Jersey, United States; University of Nevada, Las Vegas, Las Vegas, Nevada, United States

## Abstract

As the adult population ages, chronic multimorbidity is estimated to increase by over 90%, leading to decreased quality of life. Palliative care supports chronic disease management and increases quality of life. Newer subspecialties of palliative care (e.g., neuro-palliative care and geri-palliative care) holistically address disease management and provide interventions to older adults with chronic multimorbidity and cognitive concerns to address their unique health needs in an inpatient setting. This study describes the development of PAL MIND, a clinical tool that uses a combination of intake and assessment screeners to comprehensively identify patients’ psychological needs during their inpatient stay as well as inform outpatient treatment next steps; additionally, the tool grows interdisciplinary understanding of psychological services available to patients. PAL MIND was developed based on a literature review of measures validated in palliative and geriatric patient samples. Intake sections include: patient demographics, biopsychosocial spiritual evaluation, risk evaluation, mental status exam, functional evaluation, and treatment communication evaluation. Assessment sections include: orientation screener, cognitive screener, mental health screener, palliative care screener, and advanced care planning screener. Next, we will pilot-test the tool in a regional inpatient hospital setting to determine the feasibility of tool delivery in an ICU setting. The tool has the potential to identify patient needs regarding psychological services and treatment preferences, which can facilitate interdisciplinary care and targeted treatment interventions for older adults with chronic and terminal diseases in inpatient settings.

